# The abundance of small mammals is positively linked to survival from nest depredation but negatively linked to local recruitment of a ground nesting precocial bird

**DOI:** 10.1002/ece3.9292

**Published:** 2022-09-11

**Authors:** Veli‐Matti Pakanen, Risto Tornberg, Eveliina Airaksinen, Nelli Rönkä, Kari Koivula

**Affiliations:** ^1^ Ecology and Genetics Research Unit University of Oulu Oulu Finland

**Keywords:** alternative prey, local recruitment, nest success, voles, wader

## Abstract

Generalist predators using small mammals as their primary prey are suggested to shift hunting alternative prey such as bird nests, when small mammals are in short supply (the alternative prey hypothesis, APH). Nest survival and survival of young individuals should be positively linked to small mammal abundance and negatively linked to predator abundance, but little information exists from survival of chicks, especially until recruitment. We test these predictions of the APH using 13 years (2002–2014) of life history data from a ground nesting shorebird breeding on coastal meadows. We use small mammal abundance in the previous autumn as a proxy for spring predator abundance, mainly of mammalian predators. We examine whether small mammal abundance in the spring and previous autumn explain annual variation in nest survival from depredation and local recruitment of the southern dunlin *Calidris alpina schinzii*. As predicted by the APH, survival from nest predation was positively linked to spring small mammal abundance and negatively linked to autumn small mammal abundance. Importantly, local recruitment showed opposite responses. This counterintuitive result may be explained by density‐dependent survival. When nest depredation rates are low, predators may show stronger numerical and functional responses to high shorebird chick abundance on coastal meadows, whereas in years of high nest depredation, few hatching chicks lure fewer predators. The opposite effects on nest and local recruitment demonstrate the diverse mechanisms by which population size variation in primary prey can affect dynamics of alternative prey populations.

## Graphical abstract

Using long‐term life history data, we show that nest survival and juvenile survival of a precocial shorebird are linked to the regional small mammal abundances during the breeding season and during the previous autumn. While these results support the alternative prey hypothesis, they also show that variation in small mammal abundance can have different impacts on different life history stages of the alternative prey.

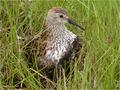

## 1 INTRODUCTION

Generalist predators, which prey on small mammals (e.g., voles) as their primary prey, are suggested to shift to hunting alternative prey such as bird nests, hares or roe deer fawns, when small mammals are in short supply (“Alternative prey hypothesis” APH, Angelstam et al., 1984; Barraquand et al., 2015; Dell'Arte et al., 2007; Kjellander & Nordström, 2003; Korpimäki et al., 1991; Reif et al., 2001). Thus, variation in abundance of the main prey of generalist predators should cause variation in depredation pressure of alternative prey species and have consequences for alternative prey populations (Angelstam et al., 1984). This prediction has been confirmed by a vast number of empirical studies (Angelstam et al., 1984; Bowler et al., 2020; Breisjøberget et al., 2018; Kjellander & Nordström, 2003; Lehikoinen et al., 2016; Tornberg et al., 2012). Importantly, not all empirical studies have found support for the APH (e.g., Ludwig et al., 2020; Reiter & Andersen, 2011) indicating potentially more complex predator prey dynamics and the need for more research.

**FIGURE 1:**
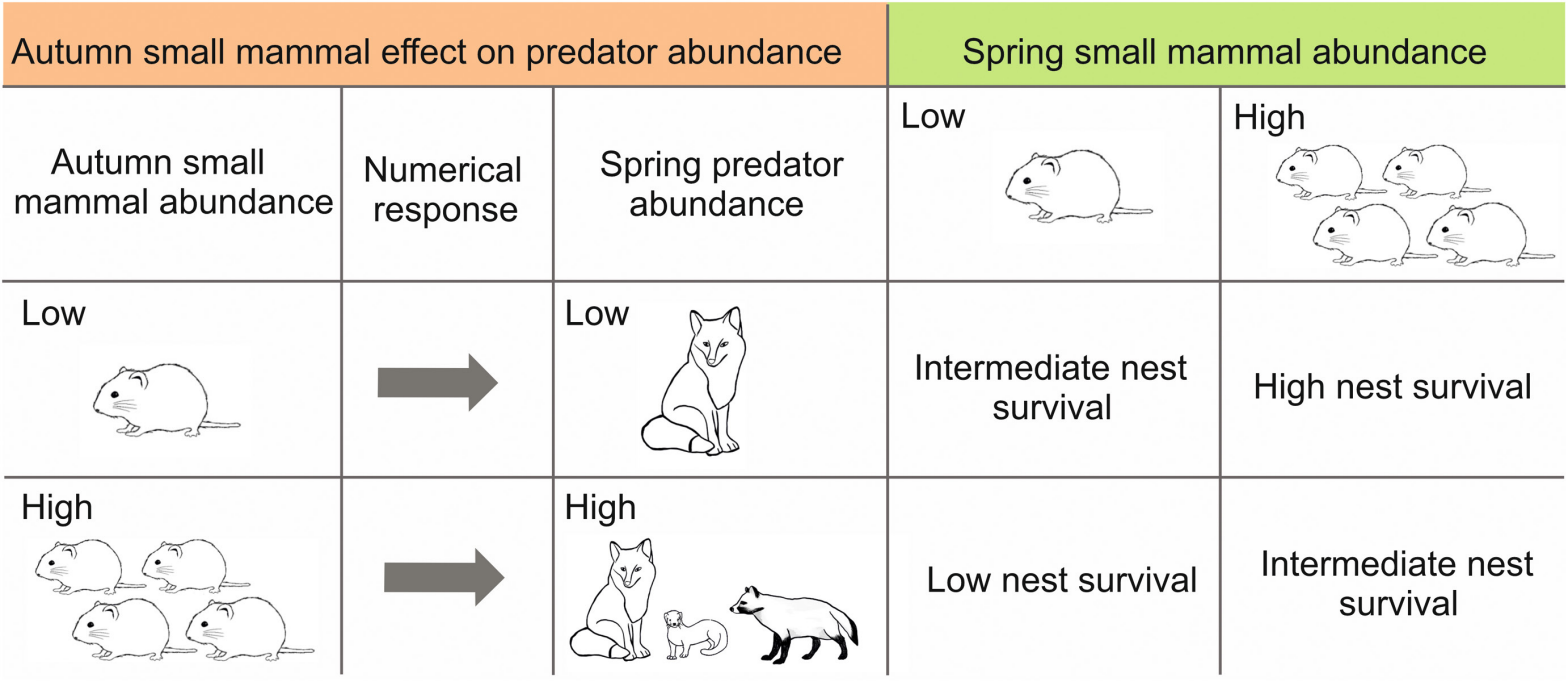
Hypothetical effects of previous autumn and spring small mammal abundances on bird nest survival. Breeding season predator abundance results from a numerical response to previous autumn small mammal abundance.

A change in population size of alternative prey results mainly from lowered reproduction when predators switch to foraging on egg and juvenile stages of the alternative prey (Breisjøberget et al., 2018; Kjellander & Nordström, 2003). Among avian alternative prey populations, this is expected to impact especially ground nesting birds, such as grouse, shorebirds and waterfowl, whose nests are vulnerable to predators and who are not the focus of depredation when small mammal populations are high (Brook et al., 2005; Iles et al., 2013; McKinnon et al., 2014; Valkama et al., 2005). Indeed, positive correlations between the number of juvenile shorebirds at nonbreeding sites and the rodent abundance at their arctic breeding sites during the breeding season when the chicks hatched provide evidence for prey switching acting on ground nesting birds (Blomqvist et al., 2002; Summers et al., 1998). This may result from increased depredation of eggs and/or young. However, evidence from survival of young (during pre‐fledging and post‐fledging phases) is scarce, especially until local recruitment, and often indirectly measured via brood size (Breisjøberget et al., 2018; Ludwig et al., 2020; Marcström et al., 1988; Schmidt et al., 2008) and results from nest survival studies are not consistent. APH predicts low nest survival when small mammal abundance is low, and high nest survival when small mammal abundance is high (Figure 1). Such relationships have been found in multiple avian studies (Bêty et al., 2001, 2002; Marcström et al., 1988; McKinnon et al., 2014; Schmidt & Ostfeld, 2008; Wegge & Storaas, 1990) but some studies find other mechanisms acting via, e.g., weather or incidental depredation with or without an aggregation of predators (numerical response) to be more important for explaining variation in nest depredation (Grendelmeier et al., 2018; Ludwig et al., 2020; Machín et al., 2019; Pöysä et al., 2016; Schmidt et al., 2008; Weiser et al., 2018).

Depredation rates are partly dependent on predator abundance (Weiser et al., 2018; Zanette & Jenkins, 2000). Hence, the relationship between depredation rates of alternative prey and small mammal abundance may not be clear when the abundance of predators varies in time. Predators show a strong numerical response to small mammal abundance, and autumnal small mammal abundance affects the number of predators present in the next breeding season by increasing winter survival of predators and by affecting investment on reproduction (Brommer et al., 2002; Korpimäki et al., 1991, 2020; Korpimäki & Norrdahl, 1991; Masoero et al., 2020). Therefore, small mammal abundance in the autumn can be a good proxy of predator abundance in the next breeding season. If the small mammal population has crashed during the previous autumn or winter, predator numbers may have also crashed due to low survival of young and adults leading to lower nest depredation rates (Figure 1). Alternatively, if the small mammal numbers have remained high, the predator population may be large and thereby nest depredation rates remain high (Figure 1). Thus, examination of the alternative prey hypothesis also warrants the consideration of predator abundance or lagged effects of rodent numbers from the previous year.

Here, we test predictions of the alternative prey hypothesis on a small ground nesting shorebird, the southern dunlin (*Calidris alpina schinzii*; hereafter dunlin) which breeds on coastal meadows in Bothnian Bay, Finland. As in many ground‐nesting bird populations, the most common cause of nest failure is nest depredation (Pakanen et al., 2011). There is substantial annual variation in predation pressure (Pakanen et al., 2011), which could be linked to the abundance of small mammals as the most commonly seen nest predators include both mammalian predators (e.g., red foxes *Vulpes vulpes*, Kaasiku et al., 2022; own observations and raccoon dogs *Nyctereutes procyonoides*; Dahl & Åhlén, 2019; own observations) and bird predators (e.g., marsh harriers *Circus aeruginosus*; Opermanis, 2004; own observations) that are known to consume small mammals. Small mammal populations in Finland also show annual fluctuation (Korpimäki et al., 2005; Sundell et al., 2004), and this variation can potentially exert varying predation pressure towards alternative prey. Importantly, small mammals are extremely rare on these coastal meadows during the breeding season (own observations). This is likely due to the fact that coastal meadow habitats in Bothnian Bay are very low and easily develop an ice cap due to recurrent winter flooding making these habitats inhospitable until the summer when new vegetation starts to grow. Furthermore, dunlins arrive to the breeding sites in late April or early May and start to breed as early as possible after the ice and snow melt, and their nests usually hatch when the meadow vegetation starts to grow in June (Pakanen et al., 2016, 2018). Small mammals nevertheless live in coastal forests and agricultural field areas that border the meadows, and hence this primary prey source exists within ca. 200–1000 m from the dunlin nest sites (own observations). The low abundance or near absence of the primary prey species (small mammals) on these coastal meadow breeding sites of the dunlin makes our study population an insightful system to test the alternative prey hypothesis because in most systems where the APH has been previously tested, the primary prey and alternative prey co‐occur in the same areas (e.g., McKinnon et al., 2014). This means that those predators that use small mammals as their primary prey must specifically travel to the coastal meadows to prey on shorebirds, instead of finding shorebirds while searching for small mammals. In this study, we use 13 years (2002–2014) of data on breeding dunlins and small mammal abundance to test whether variation in the number of primary prey (small mammals) for general predators in the spring and previous autumn explain temporal variation in nest depredation rates and local recruitment (survival from hatching until age of one years) of the dunlin.

## 2 METHODS

### 2.1 Study population and data collection

**FIGURE 2:**
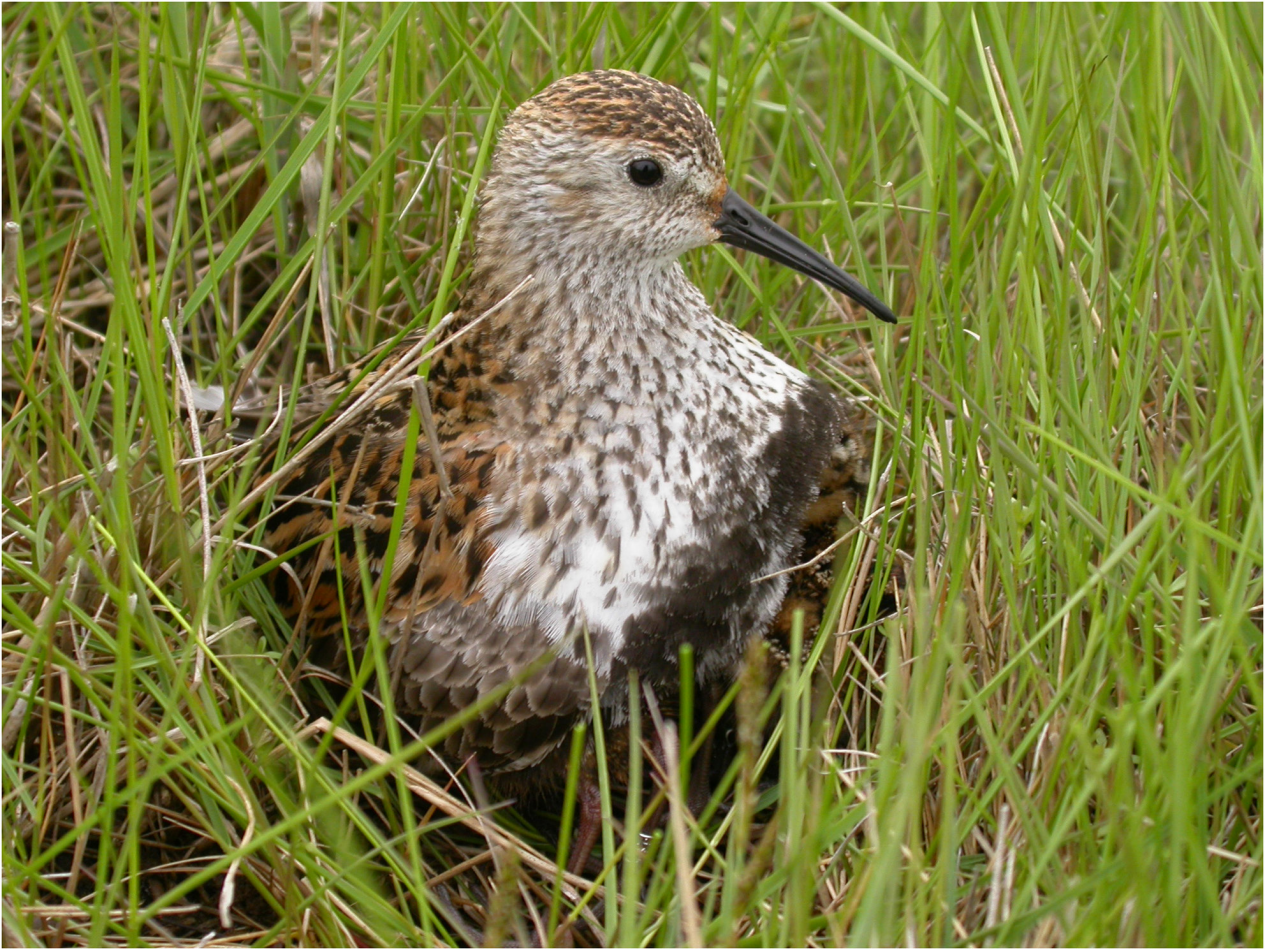
Adult dunlin (*Calidris alpina schinzii*) brooding chicks. Photo by Kari Koivula

We studied a dunlin population breeding on coastal meadows at Bothnian Bay, Finland (64° 50′ N, 25° 00′ E). We have collected individual based data from this population since 2002 (Pakanen et al., 2016). Each year, we started field work when the dunlins started to arrive and display at the breeding sites, from late April or early May, and continued until July. Field work included searching for all territories and nests and following nest fates until failure or hatching (Pakanen et al., 2011). We determined the cause of nest failure from depredation, flooding, trampling, or other causes (Pakanen et al., 2014). We estimated hatching dates on the basis of egg laying phase or incubation phase by floating the already incubated eggs (Liebezeit et al., 2007). After the eggs hatched, we ringed all chicks with metal rings (Pakanen et al., 2016; Figure 2). The rings allowed us to identify young individuals when they recruited back to the breeding population as adults. Those offspring that later returned to breed in the population were caught and ringed with a combination of color rings and they were subsequently resighted using these combinations (Pakanen et al., 2016). These data allowed us to estimate local recruitment, i.e., survival of chicks from hatching until age of one year (see below). As the breeding sites of dunlin are coastal pastures that are short‐vegetated patches among unsuitable habitat (e.g., reed beds and forest), we were able to include all of their breeding sites in our sampling. Furthermore, the population is separated by 400 km to the next dunlin population, and it is genetically differentiated from the rest of the dunlin populations breeding in the Baltic region (Rönkä et al., 2021). Thus, natal dispersal movements beyond the scale of the study area should be extremely rare, which allowed us to reliably estimate survival of chicks from hatching until age one (Pakanen et al., 2016, 2017).

### 2.2 Small mammal trapping

We monitored variation in small mammal abundance in Sanginjoki (N 65° 0′; E 25° 46′), which is situated roughly 10 km east from the city of Oulu. The trapping area is ca. 30–50 km from the study meadows, but vole abundances can be safely assumed to reflect abundances in study area because of much larger scale geographical synchrony in their population dynamics (e.g. Sundell et al., 2004). We trapped small mammals using the small quadrat method (Myllymäki et al., 1971). Each small quadrat was 15 m × 15 m in size. We placed three baited (rye bread) snaptraps roughly 1–2 meters from each corner of the small quadrat. Thus, each small quadrat had 12 snaptraps. We monitored 10 small quadrats annually during the spring (mid‐May to mid‐June; spring small mammal abundance) and autumn (late August to September; autumn small mammal abundance). Traps were monitored for 2–3 nights, and the traps were checked nightly. All small mammal species (*Microtus*, *Myodes*, and *Sorex*) were recorded. These species fluctuate synchronously (Korpimäki et al., 2005). The number of trap nights was on average 251 (SD 75) per season. It varied based on the number of days the traps were monitored. We calculated an annual index of small mammal abundance separately for spring and autumn as the number of trapped small mammals per 100 trap nights. The small quadrats were placed in abandoned field habitat (3), pine forests (1), spruce forests (3), deciduous forest (1), and young planted forest (2), which represent common habitats where small mammals live in this region.

### 2.3 Data analysis

We analyzed daily nest survival of dunlin from depredation with program MARK version 9.0 (Dinsmore et al., 2002; White & Burnham, 1999) using data from 2002 until 2014. The data included 441 nests and 5005 nest days. Depredated nests were considered as failed, whereas nests that were destroyed by other causes than depredation were considered to have survived until the estimated time of failure (mid‐point of last two observations).

We used program MARK to analyze local recruitment (survival from hatching until age one) using an age‐dependent version of the CJS model (Cormack‐Jolly‐Seber model; Lebreton et al., 1992). Here, we used birds that were ringed as hatchlings from 2002 until 2014 but included their encounter histories until 2017 to control for recruitment of individuals born in the latter years of the study. These data included 873 chicks. In the CJS model, survival probability (Φ) is corrected for the probability of recapture (*p*). We started with a general model [Φ(age1t/age2c) p(age1t/age2c)]. This model included the effect age (age: juveniles age1 vs. adults age2). Survival and recapture probabilities were constant in time for adults (age2c) but time‐dependent for juveniles (age1t; a categorical variable). This model fit the data (bootstrapping goodness of fit; *p* = .16, *ĉ* = 1.11). We were not able to reliably estimate survival in 2004 because only 9 chicks hatched. Hence, we separated the year 2004 with a separate parameter and fitted the covariates to years (2002–2003 and 2005–2014).

For both daily nest survival from depredation and local recruitment, we first fit time‐dependent (categorical year effect) models to examine temporal variation in survival and to allow estimation of the percentage of temporal variation explained by covariates (see below). We compared the time‐dependent model with a constant (intercept only) model to check for annual variation in survival. After this, we fitted models where survival was a function of linear or quadratic effects of (1) spring small mammal abundance and/or (2) previous autumn small mammal abundance. Here, spring abundance is the number of small mammals trapped per 100 nights during the same spring as the nests were followed and juveniles hatched (year t). If the generalist predators switch to hunting shorebird eggs and chicks when the small mammal abundance is low, we can expect a positive association between small mammal abundances and survival. Previous autumn small mammal abundance is the number of small mammals trapped per 100 nights during the autumn of the previous year (i.e., year *t* − 1). If the autumn small mammal abundance affects how well the predators and their offspring survive from autumn to the next breeding season, it should be linked to the amount of predators in the next breeding season, and we can expect that there is a negative association between previous autumn small mammal abundance and survival. Local recruitment includes both pre‐fledging survival (period before chicks are capable of flying) and post‐fledging survival (period after starting to fly) until age of one year. In shorebirds such as the dunlins, local recruitment reflects mostly pre‐fledging survival rather than survival during the post‐fledging period (Pakanen et al., 2021). This means that we can expect that the small mammal abundances at the breeding sites affect local recruitment.

We compared the covariate models with the intercept model to calculate support in explaining variation in daily nest survival. Furthermore, we used the analysis of deviance (ANODEV) to calculate the percentage of annual temporal variation explained by the covariate(s) as follows:

$$\frac{{\mathrm{Dev}}_{\left(c\right)}-{\mathrm{Dev}}_{\left(\mathrm{cov}\right)}}{{\mathrm{Dev}}_{\left(c\right)}-{\mathrm{Dev}}_{\left(t\right)}}$$

where Dev_(c)_ is the deviance from the constant model, Dev_(cov)_ is deviance from the covariate model and Dev_(t)_ is the deviance from the time‐dependent model (Grosbois et al., 2008, see, e.g., Oro et al., 2021).

We used the Akaike's information criterion corrected for small sample size AICc when comparing support for nest survival models and the Quasi‐AICc when comparing support for models explaining local recruitment (Burnham & Anderson, 2002). We considered models to have equal support when their difference in (Q)AICc was less than 2 units and considered model selection uncertainty by using model averaging to calculate survival estimates (Burnham & Anderson, 2002). However, if models within two AICc units from the most supported model included were more complex versions of a model with lower AICc (i.e., more parameters), we omitted them from the model averaging (Richards et al., 2011).

## 3 RESULTS

**FIGURE 3:**
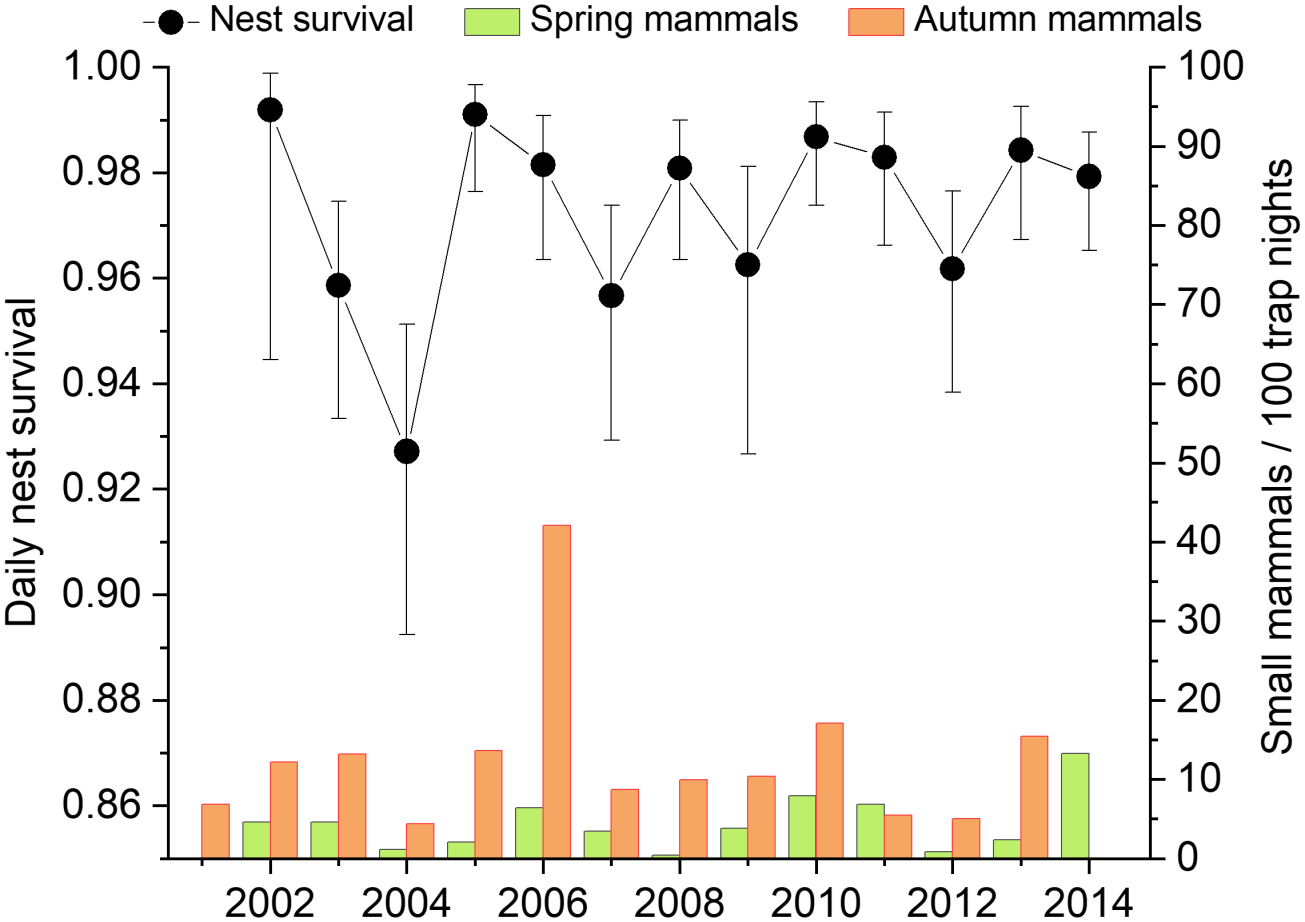
Annual variation in daily survival of dunlin nests from depredation (with 95% CI) during 2002–2014 (estimates from model A1 in Table 1) and variation in spring and previous autumn small mammal abundance.

**FIGURE 4:**
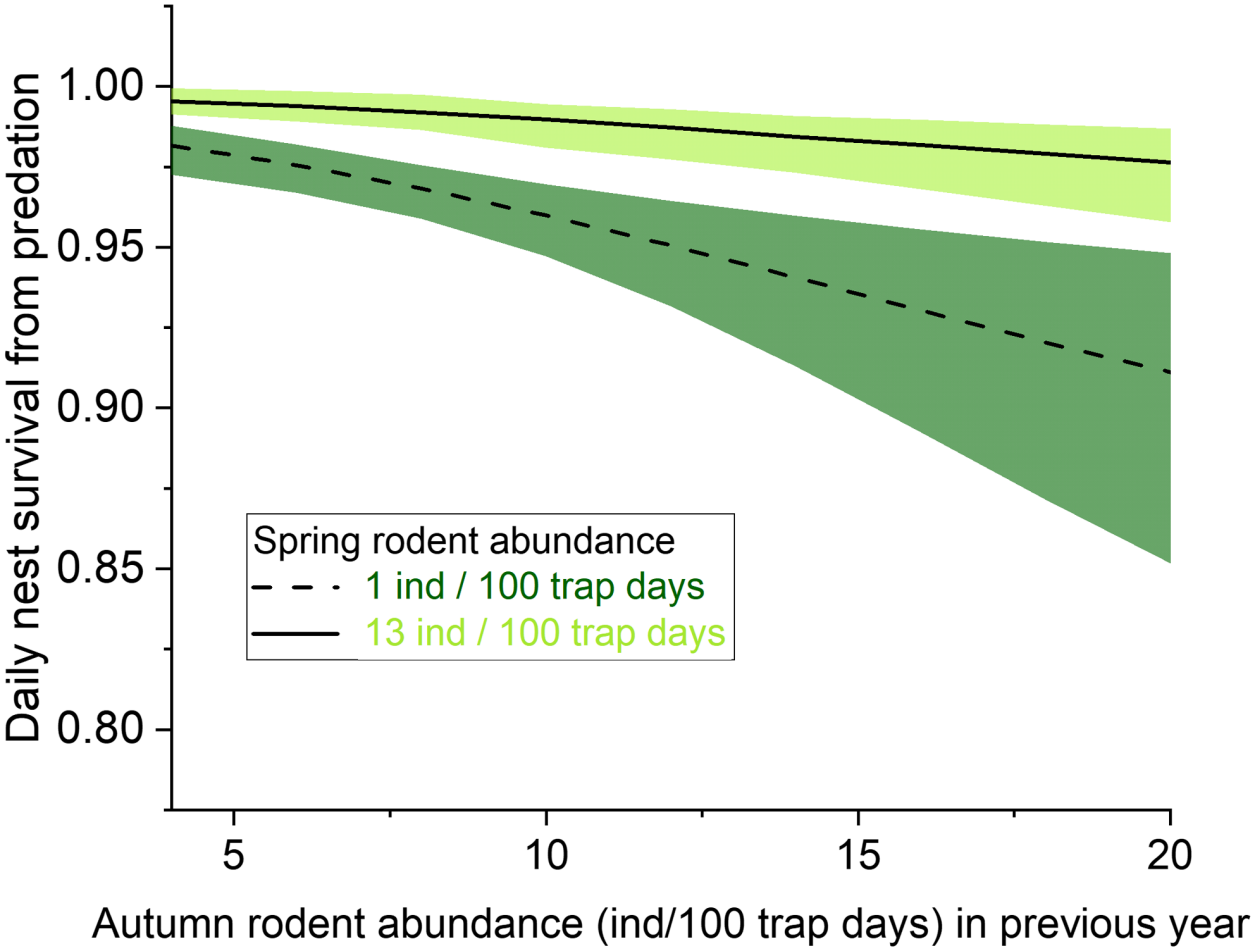
Daily nest survival of dunlin nests (with 95% CI) in relation to small mammal abundance in the previous autumn (*x*‐axis) when small mammal abundance during the breeding season (spring) is low (dashed line and dark green CI) or high (solid line and light green CI). Estimates were derived by model averaging models B2 and B3 in Table 1.

Annual variation in daily nest survival from depredation was strong (Table 1, models A1 vs. A11, ΔAICc = 24.13) varying between 0.927 and 0.992, which translate to 13–81% survival probability over the 26‐day incubation (Figure 3). Overall mean daily survival from depredation was 0.975 (SE ±0.005). The best model explaining temporal variation in daily nest survival included quadratic effects of both spring and autumn small mammal abundance, although the quadratic effect of spring small mammal abundance was weak (Table 2). Nest survival was positively linked to spring small mammal abundance and negatively linked to small mammal abundance in the previous autumn (Figure 4). Including autumn and spring small mammal abundance together clearly increased support for both variables and explained 55% of annual variation (Table 1). While the best covariate model (A2) was 18.5 AICc units more supported than the intercept model, the time‐dependent model remained the most supported model suggesting other sources of variation on annual values (Table 1).

**TABLE 1:** Models explaining variation in daily dunlin nest survival from depredation during 2002 to 2014

#	Model	AICc	ΔAICc	w	k	Deviance	%
A1	Year	795.58	0.00	0.920	13	769.51	
A2	Spring + Spring2 + Autumn + Autumn2	801.26	5.68	0.054	5	791.25	55
A3	Spring + Autumn + Autumn2	802.84	7.26	0.024	4	794.83	47
A4	Spring + Spring2 + Autumn	809.75	14.16	0.001	4	801.74	33
A5	Spring + Autumn	810.47	14.88	0.001	3	804.46	27
A6	Spring*Autumn	812.45	16.87	0.000	4	804.45	28
A7	Spring	815.87	20.28	0.000	2	811.86	12
A8	Autumn	816.53	20.95	0.000	2	812.53	11
A9	Spring + Spring2	816.80	21.21	0.000	3	810.79	14
A10	Autumn + Autumn2	818.22	22.64	0.000	3	812.22	11
A11	Intercept	819.71	24.13	0.000	1	817.71	

*Note*: Spring = spring small mammal index individuals/100 trap nights; autumn = previous autumn small mammal index individuals/100 trap nights, 2 = quadratic effect; intercept = constant model; year = annual variation; k = number of parameters; w = Akaike weight; AICc = Akaike's information criterion corrected for small sample size; ΔAICc = difference in AICc to best model, % percent of temporal variation explained by the covariate model.

**TABLE 2:** Regression coefficients of the best covariate model (model A2) explaining temporal variation in daily nest survival from depredation.

Parameter	Coefficient	SE	CI−	CI+
Intercept	4.3830	0.3637	3.6702	5.0958
Spring	0.2653	0.0795	0.1095	0.4211
Spring2	−0.0109	0.0057	−0.0220	0.0003
Autumn	−0.1808	0.0490	−0.2768	−0.0849
Autumn2	0.0031	0.0010	0.0012	0.0050

*Note*: Spring = spring small mammal index individuals/100 trap nights; autumn = previous autumn small mammal index individuals/100 trap nights; 2 = quadratic effect.

**FIGURE 5:**
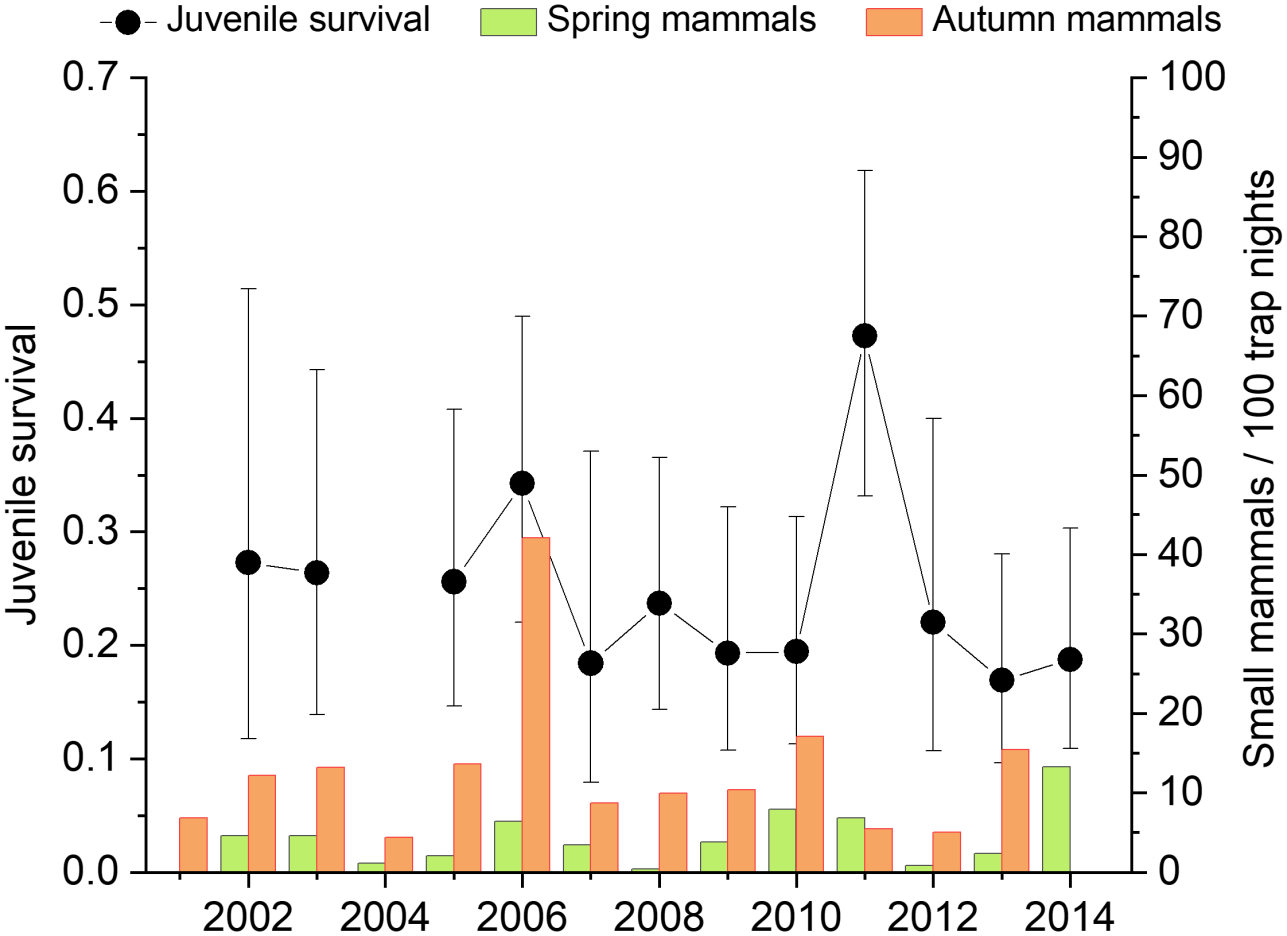
Annual variation in local recruitment of dunlin chicks (with 95% CI) during 2002–2014 (estimates from model B10 in Table 3) and variation in spring and autumn small mammal abundance.

**FIGURE 6:**
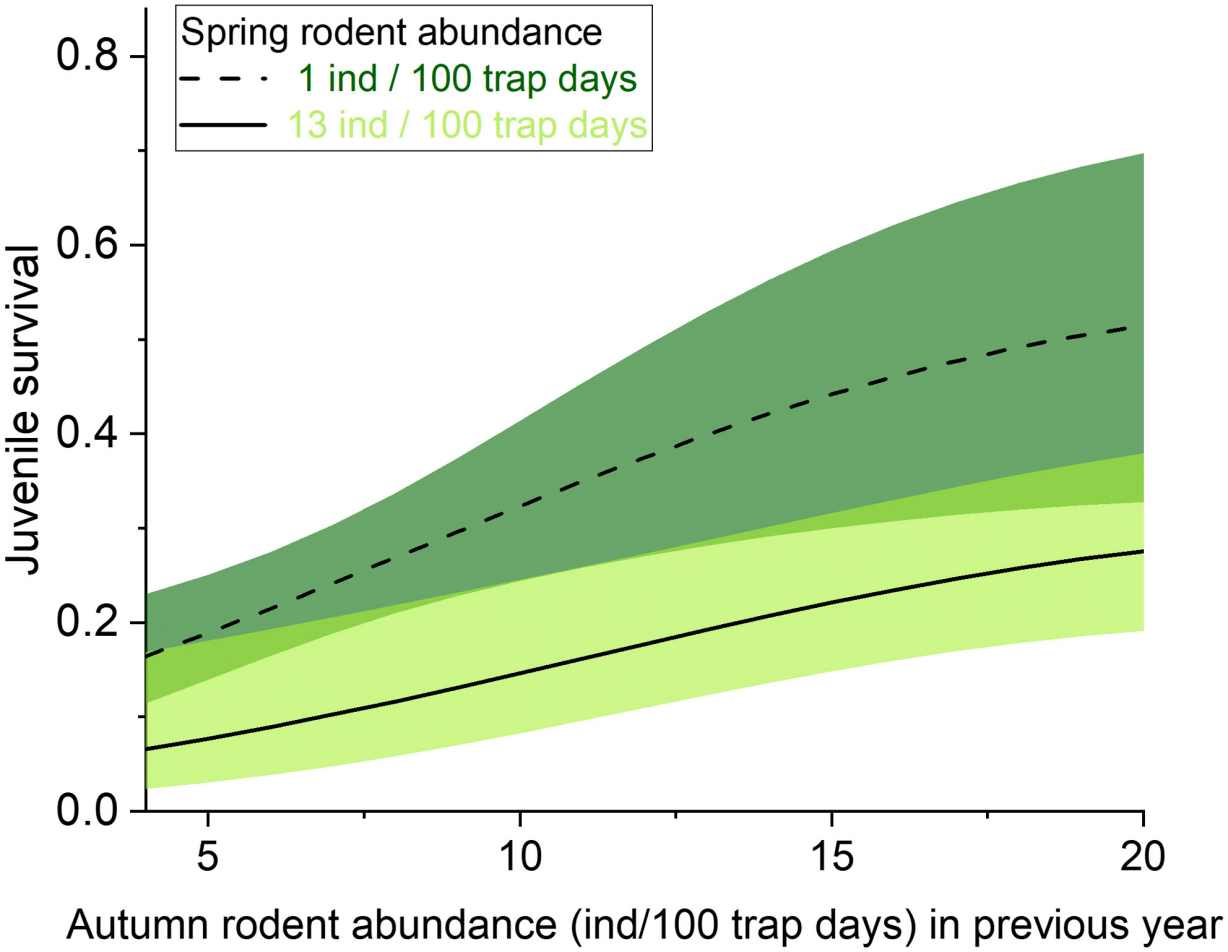
Local recruitment of dunlin chicks (with 95% CI) in relation to small mammal abundance in the previous autumn (*x*‐axis) when small mammal abundance during the breeding season (spring) is low (dashed line and dark green CI) or high (solid line and light green CI).

There was no support for strong annual variation in local recruitment (model B5 vs. model B10, Table 3). Mean survival from hatching until age one was 0.236 (SE 0.025). Annual estimates from the time‐dependent model varied between 0.169 and 0.472 but the confidence intervals of annual estimates were wide (Figure 5). The best model explaining this variation in survival suggested a quadratic effect of small mammal abundance in the previous autumn and a linear effect of spring small mammal abundance being 4.06 QAICc units better than the intercept model (Table 3). This model predicted a negative effect of spring small mammal abundance and positive effect of previous autumn small mammal abundance (Table 4; Figure 6). The second‐best model included a quadratic effect of spring small mammal abundance, but inclusion of this parameter did not increase model support. Support for linear effects of previous autumn and spring small mammal abundance were low (models B7 and B8 in Table 3).

**TABLE 3:** Models explaining variation in local recruitment of dunlin from 2002 to 2014

#	Model	QAICc	ΔQAICc	w	k	QDeviance	%
B1	Spring + Autumn + Autumn2	2008.22	0.00	0.387	8	1992.11	50
B2	Spring + Spring2 + Autumn + Autumn2	2008.75	0.53	0.297	9	1990.61	57
B3	Autumn + Autumn2	2010.59	2.37	0.118	7	1996.50	
B4	Spring + Spring2	2011.83	3.61	0.064	7	1997.75	28
B5	Intercept	2012.28	4.06	0.051	5	2002.24	22
B6	Spring + Spring2 + Autumn	2013.82	5.60	0.023	8	1997.72	22
B7	Autumn	2013.92	5.70	0.022	6	2001.86	2
B8	Spring	2014.20	5.98	0.019	6	2002.14	0
B9	Spring + Autumn	2015.92	7.70	0.008	7	2001.84	2
B10	Year	2016.45	8.24	0.006	17	1981.99	
B11	Spring*Autumn	2017.59	9.37	0.004	8	2001.48	4

*Note*: Spring = spring small mammal index individuals/100 trap nights; autumn = previous autumn small mammal index individuals/100 trap nights, 2 = quadratic effect; intercept = constant model; year = annual variation; k = number of parameters; w = Akaike weight; QAICc = quasi‐Akaike's information criterion corrected for small sample size; ΔQAICc = difference in QAICc to best model, % percent of temporal variation explained by the covariate model. The survival models include an age effect (two classes) and a separate parameter for 2004. Recapture probability model structure includes the intercept and age (two classes), i.e., p(age).

**TABLE 4:** Regression coefficients of the best model (model B1) explaining temporal variation in local recruitment.

Parameter	Coefficient	SE	CI−	CI+
Intercept	1.522	0.116	1.295	1.749
Age	−3.742	0.417	−4.560	−2.923
Year 2004	−13.926	1113.011	−2195.428	2167.575
Spring	−0.082	0.039	−0.159	−0.005
Autumn	0.196	0.062	0.073	0.318
Autumn2	−0.004	0.001	−0.007	−0.001

*Note*: Spring = spring small mammal index individuals/100 trap nights; autumn = previous autumn small mammal index individuals/100 trap nights, 2 = quadratic effect.

## 4 DISCUSSION

We tested predictions of the alternative prey hypothesis on both nest depredation and local recruitment by combining 13 years of life history data from a small ground nesting bird, and trapping data from small mammals, the primary prey of mesopredators. Results from nest depredation supported the alternative prey hypothesis but results from local recruitment suggested an opposite pattern. Hence, our detailed analysis of reproduction from egg laying until local recruitment highlights the diverse mechanisms by which population size variation in primary prey can affect dynamics of alternative prey populations.

In support of the alternative prey hypothesis, we found that spring small mammal abundance was negatively linked to nest depredation of shorebirds. Interestingly, our results describe a new aspect on how switching of prey may occur in space. In most studies, the primary prey (small mammals) and the alternative prey occupy the same areas, and predators switch to depredating bird nests when small mammal are low in abundance (Bêty et al., 2001; Marcström et al., 1988; McKinnon et al., 2014). However, small mammals are extremely rare at the coastal meadows during the dunlin breeding season (see Section ‘1’). Nevertheless, we found that nest depredation of dunlins increased when small mammals were regionally low in abundance. This suggests that predators shift foraging habitats on the basis of prey availability (e.g., Gese et al., 1996). In Finland, small mammals are mainly depredated by mammalian predators, owls, and hawks (e.g., Korpimäki et al., 1991; Sundell et al., 2004). When small mammals are low in numbers, the lack of food may cause a shift in predation pressure towards the coastal meadows from the surrounding areas. Generalist mammalian predators may be the key factor in causing this variation by consuming nests themselves but also by facilitating access of avian predators to the breeding sites. When the nests of larger shorebird species (lapwing *Vanellus vanellus*, Eurasian curlew *Numenius arquata* and black‐tailed godwits *Limosa limosa*) survive, these species provide shelter for smaller shorebird species by deterring avian predators such as corvids and birds of prey (e.g., Elliot, 1985). However, in low small mammal years, stronger movement of generalist mammalian predators such as red foxes and raccoon dogs to the coastal meadows in search of food, likely leads into depredation of nests of the larger wader species (Seymour et al., 2003). Consequently, avian nest predators such as marsh harriers and corvids will likely have better access to coastal meadows and further worsen depredation rates in years of low small mammal abundance.

Nest survival was also negatively linked to small mammal abundance in the previous autumn which we used as a proxy for predator abundance during the spring breeding season. This is in line with depredation risk being dependent on the ratio between small mammals and predators (Tornberg et al., 2011). Our results suggest that predators, which forage also on shorebird nests, show a numerical response to small mammal abundance in the previous autumn, and the peaks in small mammals likely inflict lagged long‐term consequences to nest success (Bêty et al., 2002). Small mammal abundance during previous autumn received more support in explaining nest depredation than spring small mammal abundance. In addition, the inclusion of autumn small mammal abundance was important for finding the impact of spring mammal abundance on nest survival. These results, therefore, warrant further studies where predator abundance is considered when testing the alternative prey hypothesis (see e.g., McGuire et al., 2020; Weiser et al., 2018).

Our best models explained 55% of annual variation in nest survival. The unexplained part of temporal variation in the depredation of nests can be linked to multiple processes. For example, annual variation in the number of breeding larger wader species that deter avian predators from the breeding sites (e.g., lapwings, see above) may cause annual variation in nest depredation of smaller species such as the dunlin. Furthermore, predators that specialize in small mammals can show varying behavior, and their population sizes can be affected by conditions during the annual cycle such as spread of diseases, competition or predation that that may not linked to small mammal abundance. Finally, depredation by predator species that do not specialize in small mammals when they are abundant (e.g., common gulls *Larus canus*) can create further variation.

Intriguingly, we found that small mammal abundance in the spring and autumn caused opposite effects on local recruitment compared with nest depredation. Survival of chicks from hatching until age one was lower when spring small mammal abundance was high and small mammal abundance in the previous autumn was low. Our result is similar to Ludwig et al. (2020), who reported increased depredation of red grouse (*Lagopus lagopus scotica*) chicks in years with high vole abundance. Such a pattern could be explained by the apparent competition hypothesis, i.e., incidental depredation of chicks by predators that were after small mammals (Grendelmeier et al., 2018; Mckinnon et al., 2013). However, in our case this is unlikely because small mammals are very rare at coastal meadows (see above). Instead, we hypothesize that depredation of juveniles could be density dependent (Gunnarson et al., 2006). In years when nest survival of shorebirds is low, there are very few shorebird broods, and predators will not have such a strong response to them. However, in years when nest success is high and dunlin and other shorebird chicks are more abundant, predators may show both a numerical response via aggregation to the breeding sites from other sites and a functional response to an increasing food source (Gilg et al., 2006). Assuming the same amount of initiated nests, there would be a six‐fold difference in the number of chicks present when nest survival was at the maximum we measured (81%) versus the minimum (13%). Generalist predators will use the most profitable prey and can quickly learn to use an abundant food source (Panzacchi et al., 2008). Furthermore, juvenile shorebirds are often depredated by opportunistic avian predators (marsh harriers, corvids, common gulls and arctic skuas *Stercorarius parasiticus*). The nature of the breeding sites, i.e., distinct patches that are surrounded to a large degree by forest and reedbeds, may facilitate this pattern, especially when broods of most shorebird species aggregate to the shoreline.

We show that variation in nest success of a ground nesting shorebird is linked to the abundance of small mammals. This link may be formed by generalist predators switching to alternative prey, such as shorebird nests, when small mammals are low in abundance. If this is the case, the observed pattern has important conservation implications as many ground nesting bird species, including a number of shorebirds, suffer from increased nest depredation and are declining in numbers (e.g., Kaasiku et al., 2022; Kubelka et al., 2018; McMahon et al., 2020; Rönkä et al., 2006). This results in part from an increase in the number of generalist predators, especially alien species that potentially cause more severe effects on avian prey populations than native predators (Dahl & Åhlén, 2019; Krüger et al., 2018; Nordström et al., 2003; Salo et al., 2007). Importantly, these generalist species are opportunistic, and consequently, their populations do not decline strongly following the crash of small mammal abundance. Hence, these predators have the potential to exert constantly high predation pressure. In this risky environment, peak years in small mammal abundance are extremely valuable for the ground nesting bird populations as they provide temporary relief from nest depredation.

## AUTHOR CONTRIBUTIONS

**Veli‐Matti Pakanen:** Conceptualization (lead); data curation (equal); formal analysis (lead); funding acquisition (equal); investigation (equal); methodology (lead); project administration (equal); resources (equal); supervision (equal); writing – original draft (lead); writing – review and editing (equal). **Risto Tornberg:** Conceptualization (equal); data curation (equal); formal analysis (equal); investigation (equal); methodology (equal); project administration (equal); resources (equal); supervision (equal); writing – review and editing (equal). **Eveliina Airaksinen:** Conceptualization (equal); formal analysis (equal); methodology (equal); resources (equal); writing – review and editing (equal). **Nelli Rönkä:** Data curation (equal); methodology (equal); resources (equal); writing – review and editing (equal). **Kari Koivula:** Conceptualization (equal); data curation (equal); formal analysis (equal); funding acquisition (equal); investigation (equal); methodology (equal); project administration (equal); resources (equal); supervision (equal); writing – review and editing (equal).

## CONFLICT OF INTEREST

The authors declare that they have no conflict of interest.

## Data Availability

Data are available from the Dryad Digital Repository: <https://doi:10.5061/dryad.tx95x6b1q>.
